# Complete genome sequence of bacteriophages infecting *Escherichia*, *Enterobacter*, and *Pseudomonas* isolates

**DOI:** 10.1128/mra.01345-24

**Published:** 2025-04-01

**Authors:** Aubrey Joy P. Tejada, Michael Angelou L. Nada, Ruth Antoinette D. Chin, Janna Ysabelle O. Casidsid, Joseph B. Ancla, Marel Jan G. Joloro, Mark Christian C. Reterta, Anton Roi G. Collado, Sharmen C. Berlin, Arra B. Asejo, Nikka Mae R. Yadao, Virgilio P. De Paz, Ursela G. Bigol, Rommel J. Gestuveo

**Affiliations:** 1 Department of Science and Technology, Industrial Technology Development Institute 340935, Taguig City, Philippines; 2 Department of Science and Technology, Science Education Institute, Taguig City, Philippines; 3 Department of Science and Technology, S&T Fellows Program, Taguig City, Philippines; Department of Biology, Queens College, Queens, New York, USA

**Keywords:** phage therapy, genome sequencing, antimicrobial resistant bacteria, clinical isolates, bacteriophages, Philippines

## Abstract

We reported six new species of bacteriophages belonging to the genus *Drulisvirus*, *Mosigvirus*, *Kayfunavirus*, and *Kagunavirus*, with a shared genome similarity of 77.2% to 94.5%. Seven isolates are suitable candidates for phage therapy, thereby expanding our knowledge about biocontrol alternatives against infections caused by multidrug-resistant bacteria.

## ANNOUNCEMENT

The spread of antimicrobial resistance (AMR) raises public health concerns, rendering bacteria immune to antibiotics. Phage therapy is being explored as an alternative approach to combat AMR. Thus, phage identification and characterization through laboratory assays and genome sequencing are imperative to determine suitable candidates for phage therapy (1). Here, we report the complete genome of eight bacteriophages infecting selected bacterial isolates.

Wastewater samples (1 L) from various locations (Table 1) were collected, centrifuged, and filtered sequentially using 0.45 µm and 0.22 µm membrane filters. Direct isolation was used to isolate the *Escherichia* phage, VIPECOMC06, and the *Pseudomonas* phage, VIPPAEUMC02; otherwise, enriched isolation was utilized. Briefly, 20 mL of filtrate in 20 mL double-strength Luria-Bertani broth (HiMedia #M1151) containing 1 mM CaCl_2_ was inoculated with target bacterial host (1 mL) (Table 1) for 24 hr (37℃, 160 rpm). Both methods employed double-agar overlay; single plaques were picked after 24 hr, purified for at least three rounds, and propagated to obtain high-titer phage lysates (Table 1) (2, 3).

**TABLE 1:** Genome characterizations of the *Escherichia*, *Enterobacter*, and *Pseudomonas* phages

Phage name	*Enterobacter* phage vB_ VIPECLMCO6	*Escherichia* phage vB_ VIPECOMC04	*Escherichia* phage vB_ VIPECOMC06	*Escherichia* phage vB_ VIPECOTH07	*Pseudomonas* phage vB_ VIPPAETPH1	*Pseudomonas* phage vB_ VIPPAEUMC02	*Escherichia* phage vB_ VIPECOTPH05A^*a*^	*Escherichia* phage vB_ VIPECOTPH05B^*a*^
Sampling location	Masalasa Creek	Masalasa Creek	Masalasa Creek	Tertiary Hospital	Tarlac Provincial Hospital	De La Salle Medical and Health Sciences Institute	Tarlac Provincial Hospital	Tarlac Provincial Hospital
Sampling coordinates	15°28′33″ N 12°35′42″ E	15°28′33″ N 12°35′42″ E	15°28′33″ N 12°35′42″ E	14°34′39″ N 120°59′08″ E	15°28′28″ N 120°35′09″ E	14º19’40” N 120º56’32” E	15°28′28″ N 120°35′09″ E	15°28′28″ N 120°35′09″ E
Propagation host	*Enterobacter cloacae* OMCS-22–1551	*Escherichia coli* OMCS-21–1589B	*Escherichia coli* ATCC 11229	*Escherichia coli* OMCS-22–1338	*Pseudomonas aeruginosa* OMCS-21–1583	*Pseudomonas aeruginosa* ATCC 15442	*Escherichia coli* OMCS-21–1589B	*Escherichia coli* OMCS-21–1589B
Isolation method	Enriched	Enriched	Direct	Enriched	Enriched	Direct	Enriched	Enriched
Phage titer (PFU/mL)	3.07 × 10^10^	2.6 × 10^10^	4.5 × 10^9^	6.2 × 10^10^	2.13 × 10^9^	1.0 × 10^10^	2.6 × 10^10^	2.6 × 10^10^
No. of raw reads	1,292,346	1,713,847	1,405,721	1,322,377	1,389,816	2,388,688	1,377,052	1,377,052
No. of cleaned reads	431,893	1,371,138	1,171,304	1,013,834	867,728	751,771	183,193	183,193
No. of subsampled reads	150,000	5,000	200,000	30,000	300,000	300,000	100,000	100,000
Genome size (bp)	40,245	45,658	166,719	44,205	66,340	92,031	45,476	44,015
GC content (%)	51.2	50.7	37.5	50.8	55.6	49.3	50.9	53.8
Mean coverage (×)	1554	36	112	80	1726	1297	157	428
No. of CDS	50	76	264	76	93	172	73	61
No. of CDS with predicted functions	32 (64%)	40 (53%)	155 (59%)	38 (50%)	36 (39%)	53 (30%)	34 (47%)	28 (46%)
No. of tRNAs	1	0	1	0	0	14	0	0
Lifestyle prediction	Lytic	Lytic	Lytic	Lytic	Lytic	Temperate	Lytic	Lytic
Temperate marker genes	Absent	Absent	Absent	Absent	Absent	Cro gene	Absent	Absent
Presence of ARGs^*b*^	Absent	Absent	Absent	Absent	Absent	Absent	Absent	Absent
Presence of virulence genes	Absent	Absent	Absent	Absent	Absent	Absent	Absent	Absent
Closest relative (accession number)	*Enterobacteria* phage EcoDS1 (EU734172)	*Escherichia* phage vB_EcoS_fPoEco01 (NC_073317)	*Shigella* phage phi25-307 (MG589383)	*Escherichia* phage vB_EcoS_XY1 (NC_073325)	*Pseudomonas* phage vB_PaeM_LS1 (MG897799)	*Pseudomonas* phage vB_VIPPAEUMC01 (OQ721915)	*Escherichia* phage vB_EcoS_fFiEco02 (NC_073316)	*Klebsiella* phage Kp2 (KT367886)
Genome similarity (%)	77.2	79.5	94.5	89.2	95.6	100	81.2	77.9
Phage family^*c*^	*Autographiviridae*	*Guernseyvirinae*	*Straboviridae*	*Guernseyvirinae*	*Lindbergviridae*	*Vandenendeviridae*	*Guernseyvirinae*	*Autographiviridae*
Proposed binomial name	*Kayfunavirus rjtwin*	*Kagunavirus ohbeberoi*	*Mosigvirus lindsay*	*Kagunavirus ligaya*	*Pbunavirus LS1^d^*	*Pakpunavirus vippaeumc^d^*	*Kagunavirus malou*	*Drulisvirus working-ina*
BioProject accession no.	PRJNA1164958	PRJNA1164962	PRJNA1164964	PRJNA1164959	PRJNA1164966	PRJNA1164965	PRJNA1164961	PRJNA1164961
SRA accession no.	SRR30791123	SRR30791285	SRR30791201	SRR30791124	SRR30791126	SRR30791202	SRR30791125	SRR30791125
GenBank accession no.	PQ411323	PQ423993	PQ416618	PQ417132	PQ417133	PQ429081	PQ429079	PQ429080

^*a*^ Genomes were assembled from the same sequencing library indicating that two phages were present in the sample. Further purification using Cesium chloride (CsCl) and ultracentrifugation is needed to fully separate the two phages.

^*b*^ Antibiotic resistance genes.

^*c*^ Based on current ICTV Taxonomy (MSL v40).

^*d*^ ICTV-registered species.

DNA was extracted using the DNeasy Blood and Tissue kit (Qiagen) incubating samples at 75℃ for 5–10 min to inactivate DNAse 1/RNAse A (4) and further purified using AMPure XP beads (Beckman Coulter). To prepare the sequencing libraries, 100–500 ng of DNA were tagmented and amplified for 5 cycles using the Illumina DNA prep kit and sequenced (loading concentration: 15 pM) on the Illumina MiSeq platform following the manufacturer’s instructions. On average, 1,612,925 paired-end (251 bp) reads were generated per sample.

Read quality was assessed using FastQC v0.11.9 (5) and trimmed with Trimmomatic v0.39 (6). Subsampled reads were *de novo* assembled using SPAdes v3.15.5 (parameters: --only-assembler -k 21,33,55,77,99,127) (7). Genomes were reoriented based on terminase (terL) genes of the closest relative using SeqFu (8) followed by error correction using Pilon v1.24 (9). Genome coverage was determined using BBMap v39.06 (10) and SAMtools v1.19.2 (11). Finally, phage genomes were annotated using Prokka v1.14.6 (12) and the PHROGs (13) database. Default parameters were used for all software unless otherwise specified.

Genome size ranges from 40,245 to 166,719 bp with GC content of 37.5% to 55.6% and mean coverage of 37× to 1,726× (Table 1). All genomes were identified as “complete with direct terminal repeats” using checkV v1.4 (14). Taxonomic assignment was performed using taxmyPHAGE (15). For samples that cannot be classified using ICTV-registered genomes, the closest relatives were identified using Mash v2.3 (distance cut-off: 0.09) (16) and INPHARED (v1Sep2024) database (https://github.com/RyanCook94/inphared) (17). Reference genomes were retrieved from the NCBI nucleotide database, and a phylogenetic tree was constructed based on Genome-BLAST Distance Phylogeny using VICTOR (https://ggdc.dsmz.de/victor.php#) (18).

**Fig 1:**
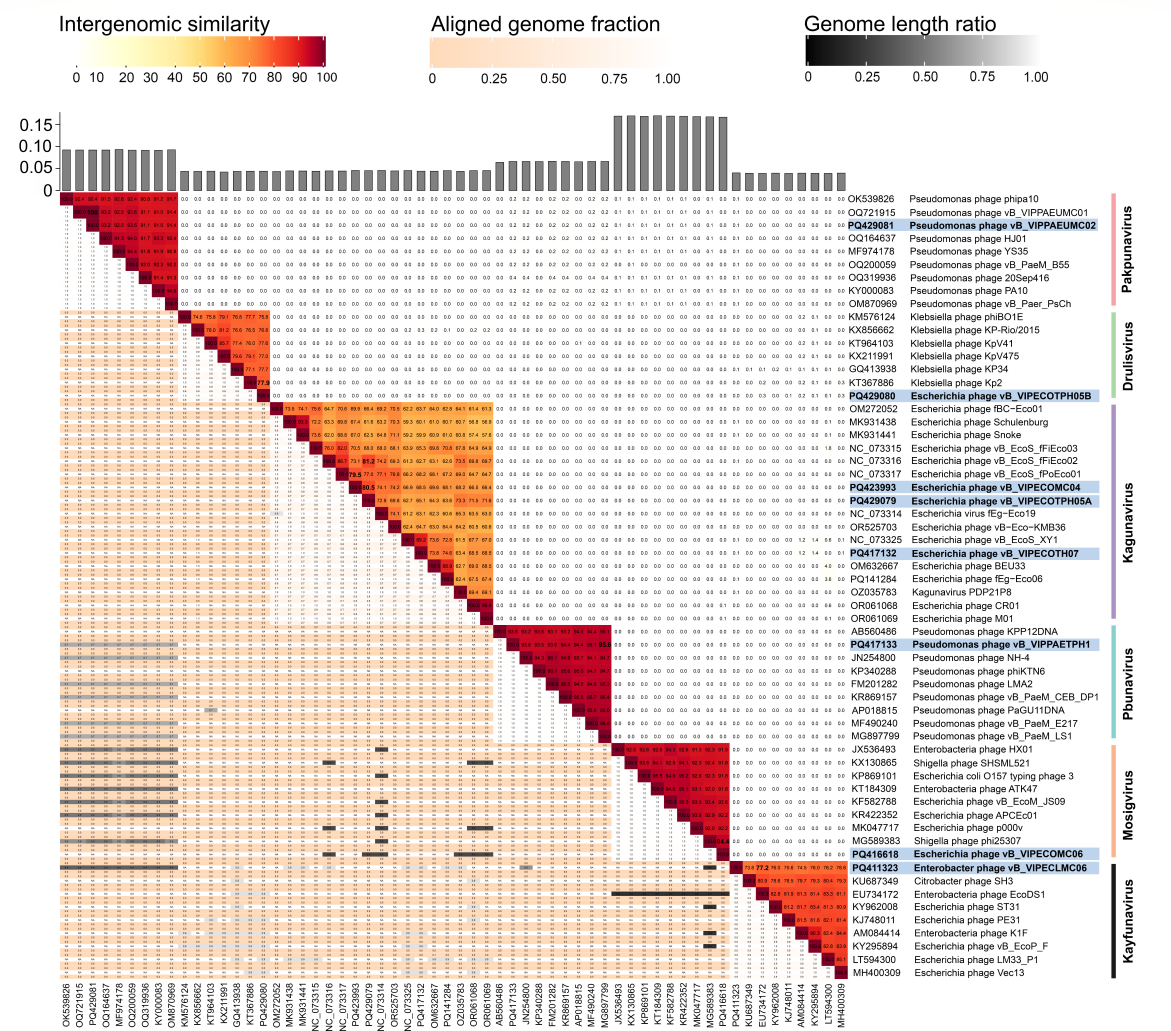
Whole-genome comparison using Virus Intergenomic Distance Calculator (VIRIDIC). The closest relatives (distance cut-off: ≤0.09) were determined with mash distance estimation using INPHARED (v1Sep2024) database, and the reference genomes of the top eight hits were downloaded from NCBI. Dark red color indicates high genome similarities, with percent similarities of the closest relative in bold. Phages with genome similarity of ≥95% are identical species. In closely related phages, a high fraction (orange to white) of the genome is aligned and is expected to have similar genome length (black to white). The phage isolates and their accession numbers are highlighted in blue.

We identified six new phage species with genome similarity of 77.2% to 94.5% using VIRIDIC v1.1 (19) (Fig. 1). These isolates belonging to the genus *Drulisvirus* (1), *Mosigvirus* (1), *Kayfunavirus* (1), and *Kagunavirus* (3) were named following the ICTV guidelines (Table 1) (20, 21). Meanwhile, the *Pseudomonas* phages VIPPAETPH1 and VIPPAEUMC02 were identical to vB_PaeM_LS1 (95.6%) and vB_VIPPAEUMC01 (100%), respectively. Finally, PhageLeads (22) predicted seven isolates with lytic lifestyle, no antibiotic resistance, and virulence genes, making them suitable for phage therapy.

## Data Availability

The raw sequences (SRA) and genome assemblies were deposited in DDBJ/ENA/GenBank under the accession numbers in Table 1.
